# Stem Cell Therapy: A Possible Role in the Treatment of Patients with Chronic Limb-Threatening Ischemia?

**DOI:** 10.14797/mdcvj.1291

**Published:** 2023-11-16

**Authors:** Annabella Olson, Bright Benfor, Eric Peden

**Affiliations:** 1 Houston Methodist DeBakey Heart & Vascular Center, Houston Methodist Hospital, Houston, Texas, US

**Keywords:** chronic limb-threatening ischemia, stem cell therapy, peripheral arterial disease

## Abstract

Chronic limb-threatening ischemia (CLTI) is a severe form of peripheral arterial disease that portends high morbidity and mortality. Patients may undergo various endovascular or open procedures with the goal of limb salvage. No-option CLTI patients represent a vulnerable population for whom conventional options have been exhausted, or anatomy precludes any attempts at revascularization, often resulting in amputation. Stem cell therapy is under investigation for these no-option CLTI patients. Regardless of revascularization technique, these patients are clinically challenging and require multidisciplinary efforts to achieve the best outcomes. Here we present a patient with unfavorable anatomy who underwent stem cell therapy injection for a nonhealing right first toe wound, and we include points to remember when considering stem cell treatment in patients with CLTI.

## Introduction

Peripheral arterial disease (PAD) places a large burden on our healthcare system even though the predicted prevalence may be significantly underestimated.^1^ Patients presenting with chronic limb-threatening ischemia (CLTI) are at an advanced stage of PAD, experiencing pain at rest and/or tissue loss that are markers of greater overall cardiovascular morbidity and mortality. While limb revascularization is a primary goal, many patients are at risk of amputation due to unfavorable anatomy or medical comorbidities that are not amenable to conventional treatment.

Stem cell therapy has been proposed as an alternate revascularization technique in patients with no-option CLTI. Bone-marrow-derived stem cells secrete angiogenic growth factors and cytokines that may improve distal perfusion.^2,3^ In small clinical trials, stem cell therapy has been found to improve amputation-free survival and reduce overall morbidity, in large part due to the improved wound healing seen in patients treated with bone-marrow derived stem cells.^4,5^ Thus far, no significant difference in adverse events has been seen in patients treated with stem cell therapy compared to placebo.5 However, whereas early angiogenesis and cell therapy studies were promising, these studies lacked sufficient control groups, and larger randomized clinical trials have yet to achieve significant benefit.^6^

Accordingly, our institution participated in a pilot, multicenter, prospective trial examining the safety and activity of bone marrow aspirate concentrate (BMAC) in patients with CLTI.^7^ Here we present an example from this study. The favorable outcomes are owed to the multidisciplinary approach required to treat patients with advanced PAD.

## Case

**Figure 1:**
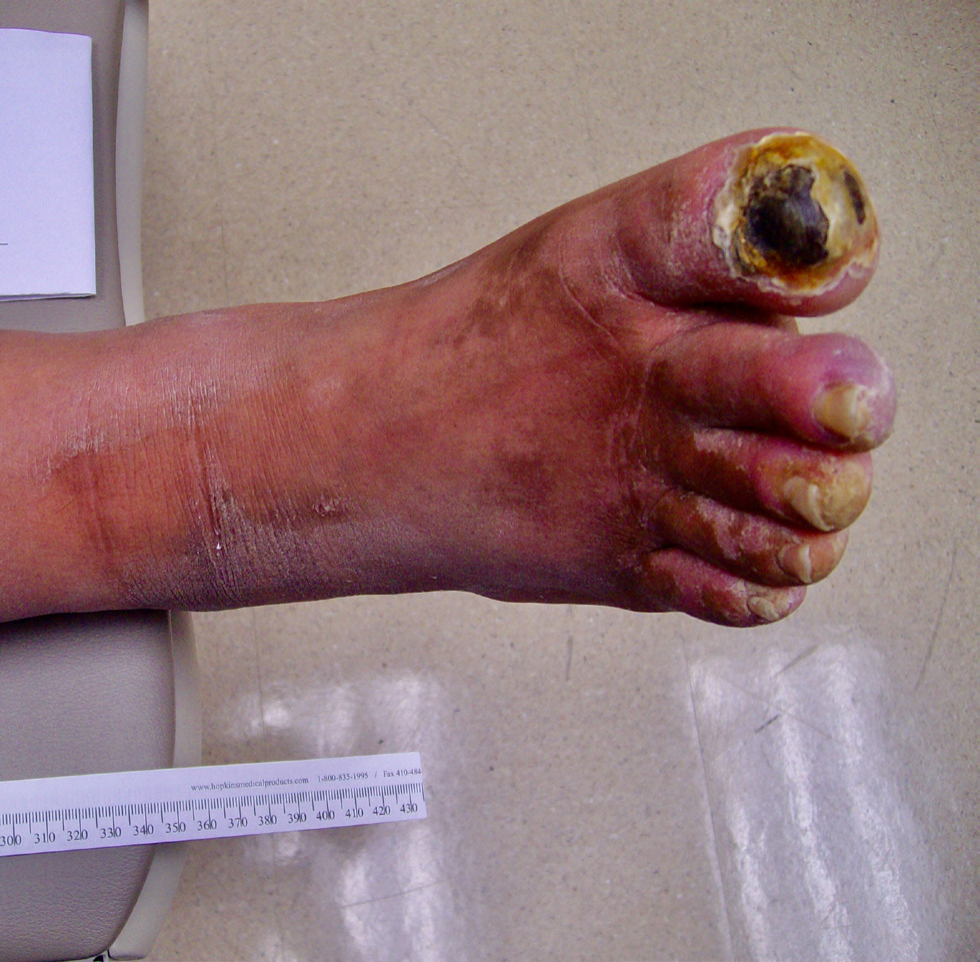
Right first toe wound prior to stem cell therapy.

A 62-year-old man with a past medical history of diabetes, hypertension, hyperlipidemia, and active smoking was referred to our vascular surgery clinic for a 6-month history of nonhealing right first toe wound. He had previously undergone right first toenail excision and debridement at an outside institution, with subsequent development of dry gangrene. He was trialed on multiple antibiotic regimens without improvement in wound healing (Figure 1). Our work-up began with noninvasive arterial studies that were normal to the level of the ankle, with decreased flow to the digits. Right lower extremity arteriogram confirmed these findings, with occlusion of the anterior and posterior tibial arteries approximately 2 cm above the ankle and only collateral vessels supplying the foot.

**Figure 2:**
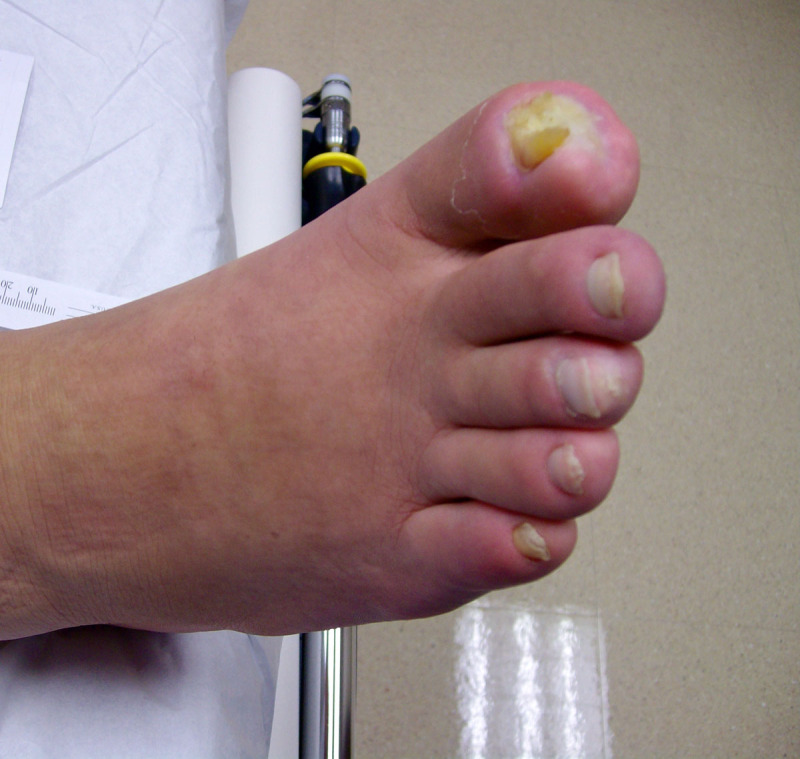
Right first toe wound 1 year after stem cell therapy.

His anatomy precluded the use of conventional revascularization procedures, and he was therefore deemed an appropriate candidate for BMAC treatment. The patient’s bone marrow aspirate was obtained and prepared using a centrifuge technique. Ultrasound-guidance was used to inject 1 mL of bone marrow aspirate near the distal patent anterior and posterior tibial arteries and in the healthy subcutaneous tissue near the right first toe wound. No adverse events were related to the procedure and the patient was discharged the same day. Approximately 1 month later, he was found to have osteomyelitis of the distal phalanx of the right first toe, confirmed on magnetic resonance imaging. He was initiated on long-term intravenous antibiotics, and after multidisciplinary discussion with the Orthopedic Surgery and Infectious Disease teams, the decision was made to continue nonoperative management. His medical comorbidities were optimized along with wound care, allowing for improved wound healing at 1 year follow-up and avoiding the need for amputation (Figure 2).

## Points to Remember

- Owing to their severe form of PAD, patients with CLTI require a multidisciplinary team approach due to their increased cardiovascular morbidity and mortality.
- No-option CLTI patients are a vulnerable population for which stem cell therapy suggests promising results.
- Through a complex process that is not completely understood, bone-marrow derived stem cells promote angiogenesis, which may allow for limb-salvage.
- Stem cell therapy for CLTI is not approved by the US Food and Drug Administration at this time and remains investigational. It appears safe and feasible, with trends towards decreased amputation rates and improved morbidity.
- Smoking cessation, medical management of comorbidities, and attention to wound care remain paramount to success even after stem cell therapy.
- Further research is needed to understand the impact of stem cell therapy for CLTI on a larger scale.

## References

[1] Aday A, Matsushita K. Epidemiology of Peripheral Artery Disease and Polyvascular Disease. Circulation Research. 2021 Jun;128:1818-1832. doi: 10.1161/CIRCRESAHA.121.318535 34110907 PMC8202714

[2] Tateishi-Yuyama E, Matsubara H, Murohara T, et al. Therapeutic angiogenesis for patients with limb ischaemia by autologous transplantation of bone-marrow cells: a pilot study and a randomised controlled trial. Lancet. 2002 Aug 10;360(9331):427-35. doi: 10.1016/S0140-6736(02)09670-8 12241713

[3] Yoder MC. Human endothelial progenitor cells. Cold Spring Harb Perspect Med. 2012 Jul;2(7):a006692 22762017 10.1101/cshperspect.a006692 PMC3385946

[4] Jeyaraman M, Nagarajan S, Maffulli N, et al. Stem Cell Therapy in Critical Limb Ischemia. Cureus. 2023 Jul;15(7):e41772. doi: 10.7759/cureus.41772 37575721 PMC10416751

[5] Powell RJ, Comerota AJ, Berceli SA, et al. Interim analysis results from the RESTORE-CLI, a randomized, double-blind multicenter phase ii trial comparing expanded autologous bone marrow-derived tissue repair cells and placebo in patients with critical limb ischemia. J Vasc Surg. 2011 Oct;54(4):1032-41. doi: 10.1016/j.jvs2011.04.006 21684715

[6] Annex BH, Cooke JP. New Directions in Therapeutic Angiogenesis and Arteriogenesis in Peripheral Arterial Disease. Circ Res. 2021 Jun 11;128(12):1944-1957. doi: 10.1161/CIRCRESAHA.121.318266 34110899 PMC8538391

[7] Iafrati MD, Hallett JW, Geils G, et al. Early results and lessons learned from a multicenter, randomized, double-blind trial of bone marrow aspirate concentrate in critical limb ischemia. J Vasc Surg. 2011 Dec;54(6):1650-8. doi: 10.1016/j.jvs.2011.06.118 22019148

